# Supplementary material for: Seminoma and Embryonal Carcinoma Footprints Identified by Analysis of Integrated Genome-Wide Epigenetic and Expression Profiles of Germ Cell Cancer Cell Lines
Source: PLoS One. 2014 Jun 2;9(6):e98330. doi: 10.1371/journal.pone.0098330 (PMC4041891; doi:10.1371/journal.pone.0098330)
Supplement: File S1 — ZIP file containing DMRforPairs output for significant regions. Please start from the html files. (ZIP) [file pone.0098330.s008.zip › figures/32138.pdf]

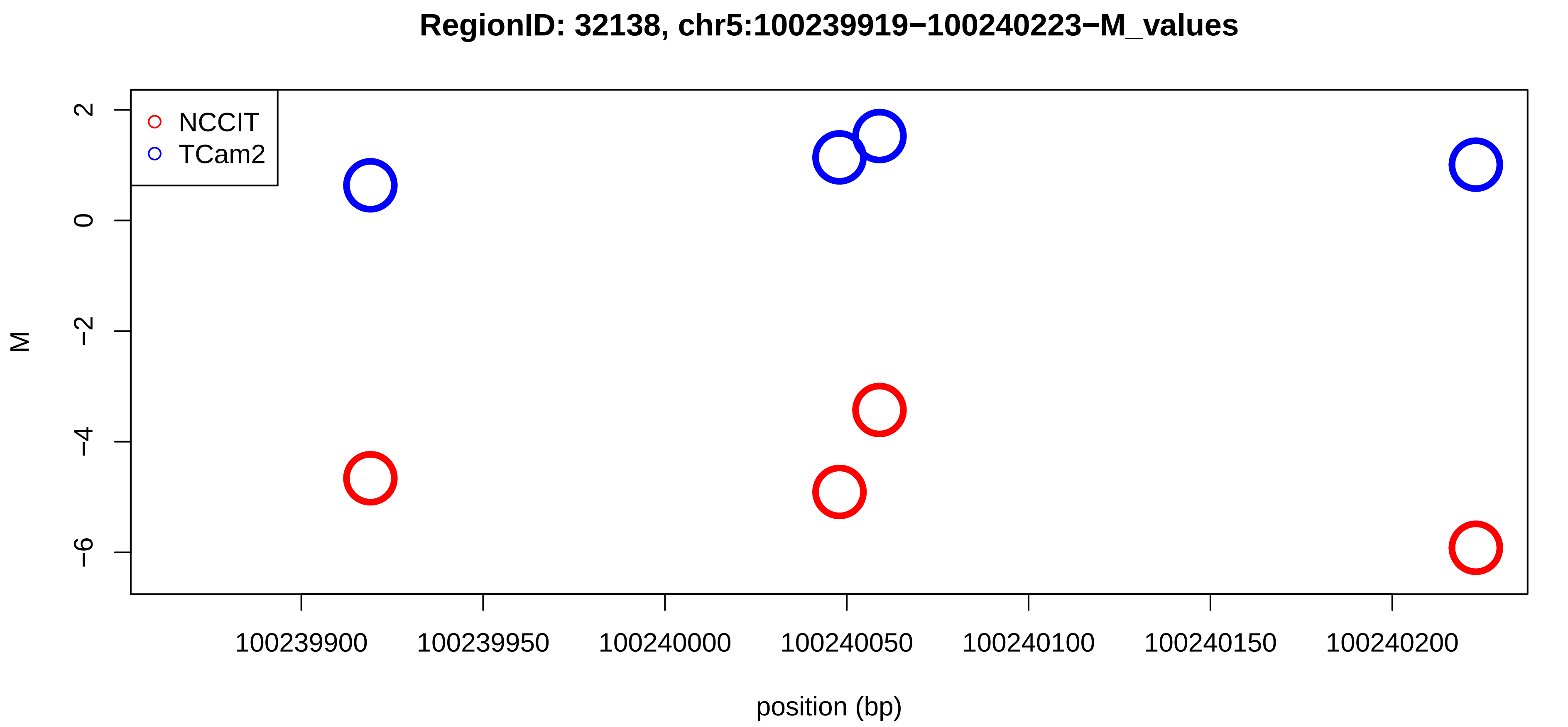
RegionID: 32138, chr5:100239919−100240223−M_values
NCCIT
TCam2
M
2
0
−2
−4
−6
100239900
100239950
100240000
100240050
100240100
100240150
100240200
position (bp)

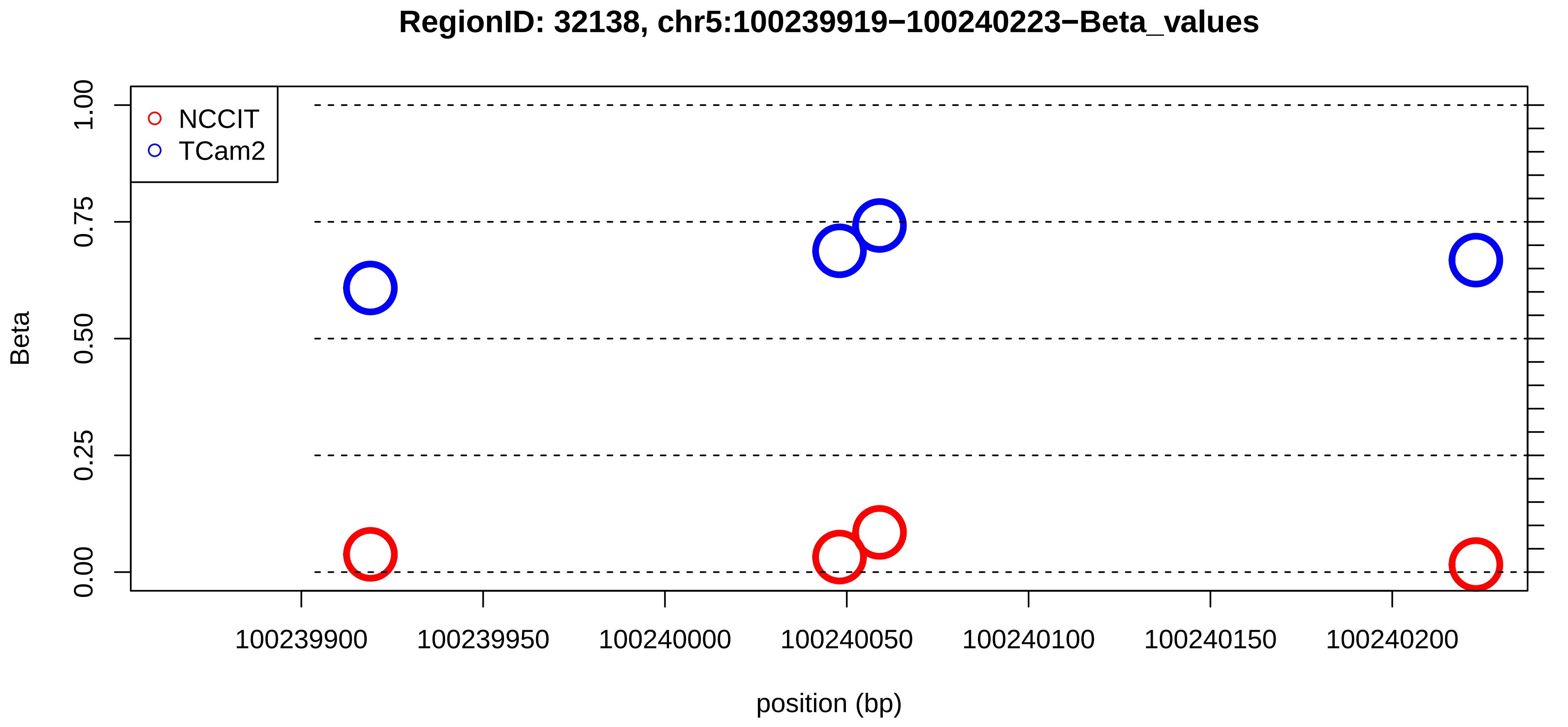
RegionID: 32138, chr5:100239919−100240223−Beta_values
NCCIT
TCam2
Beta
1.00
0.75
0.50
0.25
0.00
100239900
100239950
100240000
100240050
100240100
100240150
100240200
position (bp)
